# A Boolean network model of human gonadal sex determination

**DOI:** 10.1186/s12976-015-0023-0

**Published:** 2015-11-16

**Authors:** Osiris Ríos, Sara Frias, Alfredo Rodríguez, Susana Kofman, Horacio Merchant, Leda Torres, Luis Mendoza

**Affiliations:** Instituto Nacional de Pediatría, Laboratorio de Citogenética, Av. Insurgentes Sur 3700 C, México City, 04530 México; Programa de Doctorado en Ciencias Biológicas, UNAM, Mexico City, México; Instituto de Investigaciones Biomédicas, UNAM, Mexico City, 04510 México; Programa de Doctorado en Ciencias Biomédicas, UNAM, Mexico City, México; Facultad de Medicina/Hospital General de Mexico, Mexico City, México; C3, Centro de Ciencias de la Complejidad, UNAM, Mexico City, 04510 México

**Keywords:** Sex determination, Gonadal sex determination, Boolean model, Gene regulatory network

## Abstract

**Background:**

Gonadal sex determination (GSD) in humans is a complex biological process that takes place in early stages of embryonic development when the bipotential gonadal primordium (BGP) differentiates towards testes or ovaries. This decision is directed by one of two distinct pathways embedded in a GSD network activated in a population of coelomic epithelial cells, the Sertoli progenitor cells (SPC) and the granulosa progenitor cells (GPC). In males, the pathway is activated when the Sex-Determining Region Y (*SRY*) gene starts to be expressed, whereas in females the WNT4/ *β*-catenin pathway promotes the differentiation of the GPCs towards ovaries. The interactions and dynamics of the elements that constitute the GSD network are poorly understood, thus our group is interested in inferring the general architecture of this network as well as modeling the dynamic behavior of a set of genes associated to this process under *wild-type* and mutant conditions.

**Methods:**

We reconstructed the regulatory network of GSD with a set of genes directly associated with the process of differentiation from SPC and GPC towards Sertoli and granulosa cells, respectively. These genes are experimentally well-characterized and the effects of their deficiency have been clinically reported. We modeled this GSD network as a synchronous Boolean network model (BNM) and characterized its attractors under *wild-type* and mutant conditions.

**Results:**

Three attractors with a clear biological meaning were found; one of them corresponding to the currently known gene expression pattern of Sertoli cells, the second correlating to the granulosa cells and, the third resembling a disgenetic gonad.

**Conclusions:**

The BNM of GSD that we present summarizes the experimental data on the pathways for Sertoli and granulosa establishment and sheds light on the overall behavior of a population of cells that differentiate within the developing gonad. With this model we propose a set of regulatory interactions needed to activate either the *SRY* or the WNT4/ *β*-catenin pathway as well as their downstream targets, which are critical for further sex differentiation. In addition, we observed a pattern of altered regulatory interactions and their dynamics that lead to some disorders of sex development (DSD).

**Electronic supplementary material:**

The online version of this article (doi:10.1186/s12976-015-0023-0) contains supplementary material, which is available to authorized users.

## Background

Sex development is a complex biological process that occurs during the embryonic and fetal stages of an individual. For a better understanding sex development is divided into three consecutive steps: 1) chromosomal sex determination (CSD); 2) gonadal sex determination (GSD); and 3) phenotypic sex differentiation (PSD). CSD is established at conception when the complement of sex chromosomes, XX or XY, is received. GSD, which is the process that we analyze in this study, refers to the set of genes and their regulatory interactions that trigger the development toward testes or ovaries, underlined by a gene regulatory network [1–4]. Finally, PSD involves the development of the female and male internal and external genitalia in response to the hormones secreted by the ovaries and testes. Both male and female PSD occur in two temporal phases, the first occurs within the fetus after GSD and the second occurs during puberty [5, 6].

GSD occurs within a heterogeneously composed structure called bipotential gonadal primordium (BGP). This structure, located on the ventromedial surface of the mesonephros [5–7], is critical for sex development since it can differentiate either as testes or ovaries [8]. The BGP originates the actual gonad that is composed by *a)* the germinal cells (GCs), *b)* the steroidogenic somatic cells, such as the theca cells in ovary and the Leydig cells in testis that produce stradiol and testosterone, respectively; and *c)* the support somatic cells, including granulosa cells in ovary and Sertoli cells in testis.

Sertoli and granulosa cells originate from a common population of coelomic epithelial cells corresponding to the Sertoli progenitor cells (SPC) or granulosa progenitor cells (GPC) that migrate towards the BGP [9, 10]. In males, the SPCs start to differentiate toward Sertoli cells after 44 days of development (Carnegie-Stage 18). The mechanism involves activation of the expression of the Sex-determining Region Y gene (*SRY*) that codifies the SRY transcription factor [9, 11]. SRY associates with other transcription factors (i.e., CBX2, SF1) to regulate expression of the *SOX9* gene that positively regulates the expression of genes associated to Sertoli cells (i.e., *SOX9, FGF9, PGD2, DHH, AMH*) [2]. In females, where *SRY* is absent, GSD initiates after 49 days of development (Carnegie-Stage 20). In this case, the GPCs of coelomic origin differentiate towards granulosa cells by the action of a distinct gene regulatory pathway. Most likely, an increased amount of the transcription factor *β*-catenin up-regulates a set of downstream genes associated to granulosa, such as *FOXL2* and *RSPO1* [2, 12, 13]. Thus, the mechanism underlying GSD involves a common population of undifferentiated cells with the potential to diverge into two cell fates. The male pathway leads towards Sertoli cell fate determination and differentiation, whereas the female pathway leads to granulosa cell fate determination and differentiation.

Once differentiated, the Sertoli cells act as organizing centers, enclosing GCs to form testicular cords and secreting factors such as DHH and PDGF, which are essential for development of the fetal population of Leydig cells [14]. Granulosa cells are the female equivalent of the Sertoli cells, as they enclose GCs and secrete factors necessary for oocyte growth and maturation. The regulatory network controlling GSD and differentiation toward Sertoli or granulosa cell consists, in a broad sense, of multiple target genes, different types of RNAs, transcription factors, nuclear receptors and signaling molecules. These elements are present in undifferentiated cells and interact in a concerted way either activating or repressing target genes at the time of GSD to balance the fate toward Sertoli or granulosa cells [15–17].

The total number of genes implicated in the regulatory network of GSD of humans and mammals remains elusive, as well as their complete regulatory interactions and their effects on the process of Sertoli or granulosa cells differentiation. However, it is well known that mutations in their components underlay the so-called disorders of sex development (DSD), a series of genetic conditions characterized by anomalies in gonads as well as in internal and external genitalia. The incidence of DSDs, as estimated by the The Lawson Wilkins Pediatric Endocrine Society (LWPES) and the European Society for Pediatric Endocrinology (ESPE), is 1 in 4,500 births [18] and can be attributed to mutations in various genes of the GSD network. For example, mutations in *CBX2*, *GATA4* and *WT1* genes result in a wide range of phenotypic alterations characterized by ambiguous or female external genitalia with the presence or absence of Mullerian structures in 46,XY DSDs patients [19]. In contrast, 46,XX DSDs cause masculinization of the female fetus (normal males with no ovarian tissue) [20]. In other cases 46,XX DSD patients have a female phenotype but fail to develop ovaries, presenting instead a “streak gonad”? (streaks of connective fibrous tissue) [21].

Boolean network models (BNM) are formal tools for analyzing the structure and dynamic behavior of genetic regulatory networks. BNM are best suited for describing poorly-characterized systems with no or few kinetic details, such as the GSD network. These models represent molecular entities (genes, transcription factors and RNAs) as nodes interacting among them within a network. Each node can have only two qualitative states: 0 (OFF) and 1 (ON). The OFF state is equivalent to a below-threshold concentration or activity, which is insufficient to initiate the intended process or regulation, while the ON state is equivalent to an above-threshold concentration or activity [22]. The ON/OFF state of a node within the network is determined by a Boolean function that encompasses the known regulatory elements of the target node (transcription factors, nuclear receptors, signaling molecules). The state of these regulatory elements is updated over consecutive time steps of a simulation until the system converges to either a steady state or a cycle. BNMs describe the dynamic state of the nodes in a network by updating the state of the nodes according with the set of regulatory functions [23]. BNMs have been implemented for the analysis of developmental programs such as flower morphogenesis in *A. thaliana* [24], early cardiac development in mice [25], and expression pattern of the segment polarity genes in *Drosophila* [26] to name a few.

Despite the relatively high incidence of DSDs, their molecular basis at the level of the regulatory network remain poorly understood. Thus, we are interested in constructing a BNM of the process of gonadal sex determination with an emphasis on the regulatory elements that are present at early stages of development and control the differentiation of SCP and GCP towards Sertoli and granulosa cells, respectively, allowing us to analyze the origin of some DSDs. For in-depth reviews about the genes involved in GSD and DSDs see: [18, 19, 27, 28], as well as the list of genes and interactions in the Additional file 1 of the supplementary information.

In this study we present a BNM that describes the dynamics of the GSD regulatory network starting from the UGR until Sertoli/granulosa cells differentiation. The proposed regulatory network incorporates a large amount of published information related to functional interactions among the genes involved in this process, while the BNM of GSD describes the dynamics of the elements contained within the network under *wild-type* and mutant conditions. With the current model we explore a formal description of the functional relationships among the genes and gene products associated to GSD, and generate some predictions about the expected regulatory behavior under *wild-type* or altered conditions within elements of the UGR and elements of the bipotential gonadal primordium such as CBX2, GATA4, and WT1. Additional predictions are indicated in the female pathway where the transcription factor *β*-catenin seems to play an important role in the activation of female-specific genes (for example, *WNT4*, *RSPO1*, and *FOXL2*).

## Methods

### The network of gonadal sex determination

**Fig. 1:**
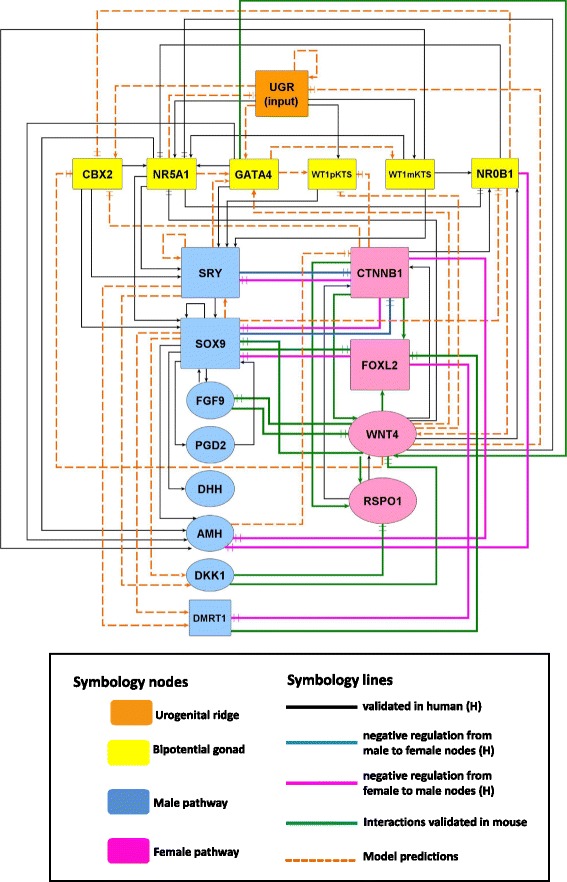
Network of Gonadal Sex Determination leading to Sertoli or granulosa cell fate commitment and differentiation. The network was inferred from reviewed experimental evidence of genes associated within the process and structured according to developmental stages in: urogenital ridge (*UGR node*), bipotential gonadal primordium (*yellow nodes*), male pathway of sex determination (*blue nodes*), female pathway of sex determination (*pink nodes*). Nodes represent genes; arrow lines denote activation; bar head lines indicate inhibition; black, blue and, pink solid lines represent validated interactions in human; green solid lines represent interactions validated in mouse; punctuated lines in orange represent model predictions

To construct the network we selected a set of genes with well-known clinical and experimental data demonstrating their association to GSD under *wild-type* and mutant conditions. The genes, depicted in a regulatory diagram (Fig. 1), include: CBX2, NR5A1, GATA4, WT1pKTS, WT1mKTS, NR0B1, SRY, SOX9, FGF9, PGD2, DHH, AMH, DKK1, DMRT1, CTNNB1, WNT4, FOXL2, RSPO1, and a special node called UGR. The interactions among these nodes are denoted by edges. We distinguish positive interactions (activation) by connecting two nodes with an arrow-head line. Negative interactions (inhibition) are denoted by connecting two nodes with a bar-head line.

The regulatory interactions among nodes were inferred, with emphasis in humans, from: (1) clinical studies of patients with DSDs, which carried mutations on sex determining genes; (2) genetic expression patterns associated to GSD (between 5th and 8th weeks of embryonic development); and (3) molecular evidence of interactions at the level of transcriptional regulation of target genes (i.e., up or down regulation of a target gene by means of protein-DNA interactions under *wild type*, mutated or transgenic constructs). Experimental evidence on mice was integrated into the network when necessary, especially in the female pathway where human information is lacking. References to clinical and experimental data can be found in the Additional file 1 of the Supplementary Information.

The network includes the special UGR node, representing the urogenital ridge, an embryonic structure precursor of the nephrogenic cord and gonads. The UGR node encompasses the following genes: *LHX1*, *LHX9*, *EMX2*, *PAX2* and, *PAX8*. Although expression of these genes is essential for growth and maintenance of the UGR, little evidence was found in human, as well as in mouse, about their specific regulatory interactions, thus these genes were grouped within the UGR node, since mutations in any of these genes impair subsequent gonadal development [29–31].

The pathway towards pre-Sertoli or pre-granulosa cells is shown in Fig. 1. The blue nodes correspond to Sertoli cell fate determination pathway and include: SRY, SOX9, FGF9, PGD2, DHH, AMH, DKK1 and DMRT1 nodes, whereas the pink nodes correspond to granulosa cell fate determination pathway including: CTNNB1, WNT4, FOXL2 and RSPO1. Notice that the granulosa cell fate determination was complemented with mice information. For example, we considered the canonical Wnt4/ *β*-catenin pathway as a key regulatory element of female nodes within the network since relative expression of *Fst*, *Gng13*, *Foxl2*, *Irx3* and, *Sp5* has been shown to be down-regulated when *β*-catenin is lost in female mice in early stages of ovarian development [32].

### The network as a Boolean model

The process of GSD is poorly characterized at the quantitative level, i.e., kinetic information regarding the interactions of the elements of this regulatory network is still lacking, therefore the implementation of the GSD network as a continuous model is, at this moment, out of reach. Given this, we decided to model the network as a discrete dynamical system so as to describe the qualitative observations that are experimentally reported. Specifically, we used a Boolean approach where every node might have one of two possible states; 1 (ON) or 0 (OFF), indicating that a given node within the network model is active or inactive, respectively.

To determine the activation state of each node in the GSD model we translated the experimental regulatory interactions into a set of Boolean functions with the use of the logical operators AND, OR and NOT (Table 1). The logical operator AND is used if two nodes named A and B are required to activate a third node named C. The logical operator OR is used if two nodes named A or B can activate, by its own, node C. The logical operator NOT is used if node A is an inhibitor of node B. Thus, the state of a given node over time is determined by the activation state of its regulators. We integrated to the model additional regulatory interactions not reported by observational or experimental studies (Table 2). These interactions were inferred from analysis of the dynamics of the Boolean model and might be considered as model predictions that deserve further experimentation to be validated. Interactions of model predictions are shown in Fig. 1 as orange dashed lines.

**Table 1:** Set of functions for the Boolean model of gonadal sex determination

UGR, UGR & ! (NR5A1 ∣ WNT4)
CBX2, UGR & ! (NR0B1 & WNT4 & CTNNB1)
GATA4, (UGR ∣ WNT4 ∣ NR5A1 ∣ SRY)
WT1mKTS, (UGR ∣ GATA4)
WT1pKTS, (UGR ∣ GATA4) & ! (WNT4 & CTNNB1)
NR5A1, (UGR ∣ CBX2 ∣ WT1mKTS ∣ GATA4) & ! (NR0B1 & WNT4)
NR0B1, (WT1mKTS ∣ (WNT4 & CTNNB1)) & ! (NR5A1 & SOX9)
SRY, ((NR5A1 & WT1mKTS & CBX2) ∣ (GATA4 & WT1pKTS & CBX2 & NR5A1) ∣ (SOX9 ∣ SRY)) & ! (CTNNB1)
SOX9, ((SOX9 & FGF9) ∣ (SRY ∣ PGD2) ∣ (SRY & CBX2) ∣ (GATA4 & NR5A1 & SRY)) & ! (WNT4 ∣ CTNNB1 ∣ FOXL2)
FGF9, SOX9 & ! WNT4
PGD2, SOX9
DMRT1, (SRY ∣ SOX9) & ! (FOXL2)
DHH, SOX9
DKK1, (SRY ∣ SOX9)
AMH, ((SOX9 & GATA4 & NR5A1) ∣ (SOX9 & NR5A1 & GATA4 & WT1mKTS)) & ! (NR0B1 & CTNNB1)
WNT4, (GATA4 ∣ (CTNNB1 ∣ RSPO1 ∣ NR0B1)) & ! (FGF9 ∣ DKK1)
RSPO1, (WNT4 ∣ CTNNB1) & ! (DKK1)
FOXL2, (WNT4 & CTNNB1) & ! (DMRT1 ∣ SOX9)
CTNNB1, (WNT4 ∣ RSPO1) & ! (SRY ∣ (SOX9 & AMH))

**Table 2:** Set of regulatory interactions inferred from analysis of the dynamics of the Boolean model, colored in orange, that deserve further experimentation to be validated

	

We performed an initial exhaustive evaluation of the dynamic behavior of the *wild type* model, simulating all possible initial activation states. Three fixed-point attractors were obtained, and we performed a search focused in finding the state transitions corresponding to both male and female pathways. To recover the *wild type* “male pathway”, we initiated the simulations with the UGR node in ON. In contrast, to created a *wild type* “female pathway”, without the SRY node, we set the UGR and WNT4 nodes as active at the beginning of simulations. Besides the *wild type* model, we simulated all possible loss and gain of function of single mutants, so as to describe alterations in activation states that might be interpreted as alterations in gene expression. Loss and gain of function single mutants were simulated by fixing the relevant node to 0 or 1, respectively. All simulations were carried out under the synchronous updating scheme with the use of BoolNet [33].

### Testing properties of the Boolean model: random networks and robustness of attractors

**Fig. 2:**
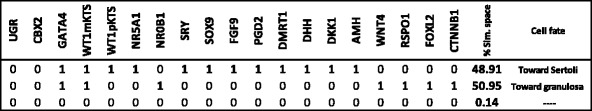
Fixed point attractors of the Boolean model of gonadal sex determination. The attractors were obtained by simulating all possible (2^19^) initial activation states. The attractor with the largest basin of attraction (50.95 %) can be interpreted as the gene expression profile observed in the somatic pre-Granulosa cells. The attractor with the second-largest basin (48.91 %) can be interpreted as the gene expression profile observed in the somatic pre-Sertoli cells. The model also presents a third attractor with a small basin covering only 0.14 % of the state space interpreted as a null attractor due to a UGR node set to zero

We performed tests by creating random networks in order to analyze the frequency of appearance of point attractors identical to those of the wild type model (Fig. 2). The tests consisted in the construction of 1000 random networks with 19 nodes each one. The number of inputs for each node in the random networks was the same as in the original model. We kept this configuration in order to be consistent with the network architecture of the model. The wild type attractors shown in (Fig. 2) were compared by performing three independent tests of 1000 random networks each one. Additionally, we tested the robustness of the attractors of the BNM with a set of 1000 perturbed copies of the network by using the *testNetworkProperties* function of BoolNet [33]. This test gives the percentage of the original attractors shown in Fig. 2 recovered after 1000 copies of the Boolean model functions randomly perturbed.

## Results

### The network of gonadal sex determination

The network was constructed with 19 nodes and 78 regulatory interactions: 42 of these interactions have been reported in humans; 12 in mice and 24 were predicted from analysis of transition states of the simulated Boolean model. The network is directed towards the male pathway if the SRY node is active. SRY leads to activation of SOX9, which in turn activates FGF9, PGD2, DMRT1, DHH, DKK1, and AMH nodes. At the same time, the female pathway is repressed by inactivating CTNNB1 and FOXL2 nodes (Fig. 1). On the contrary, the network is directed towards the female pathway in absence of SRY and when the WNT4, CTNNB1, RSPO1 and, FOXL2 nodes are active. In this case, the male pathway is repressed by CTNNB1 and FOXL2 mediated inactivation of SOX9, DMRT1 and AMH (Fig. 1).

### Predictions of the Boolean model

The current model contains 24 interactions inferred from dynamic modeling, these are predominantly related to the UGR node and the genes expressed in the bipotential gonad. Model predictions were drawn as orange dashed lines within the following nodes: UGR, CBX2, GATA, WT1mKTS, WT1pKTS, NR0B1, SRY, DMRT1, DKK1, WNT4 and CTNNB1 (Fig. 1). The model predicts that the activity of UGR depends of an activation self-loop and functions as an input to activate CBX2, GATA4, Wt1mKTS, WT1pKTS, and NR5A1 (Table 2). The scarcity of information regarding UGR function and maintenance clearly indicates that more experimental studies are necessary to understand the mechanisms of gene expression control in the BGP especially for *CBX2*, *GATA4* and *WT1*.

### Dynamic behavior of the gonadal sex determination Boolean network model

The dynamic behavior of the GSD BNM was exhaustively analyzed by starting the dynamical simulations of the system from all possible 2^19^=524288 initial states. After simulations, three fixed-point attractors where obtained (Fig. 2). The first of these attractors can be interpreted as the gene expression profile observed in Sertoli cells, the second can be interpreted as the gene expression profile observed in granulosa cells, and the third attractor, with a very small basin of attraction, might represent a disgenetic gonad without Sertoli or granulosa activity.

**Fig. 3:**
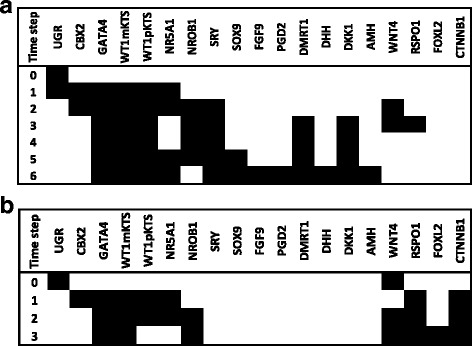
State transitions leading to a fixed point attractor corresponding to pre-Sertoli or to pre-granulosa cells. The state of the nodes over time was simulated starting from an activated UGR node (1). White and black cells represent inactive or active nodes respectively. The fixed point attractor toward pre-Sertoli is given by activation of SRY node (**a**). The point attractor is in agreement with experimental observations after six time steps, whereas the fixed point attractor toward granulosa is given by absence of SRY and activated UGR+WNT4 nodes (**b**). The point attractor is in agreement with experimental observations after three time steps

After the initial exhaustive search of the GSD attractors, we performed a search focused in finding the state transitions of both male and female pathways. In the case of 46,XY simulations, the UGR node was set to ON in the initial condition (Time step 0) to transit toward the BPG and then turning ON the SRY node leading toward the Sertoli cell attractor. Since 46,XX *wild type* females do not have *SRY* gene, we searched from all possible initial states the activation patterns that had UGR and female nodes as initial condition. From this search we found that UGR + WNT4 were the initial conditions (Time-step 0) to transit from the BPG toward the granulosa attractor. Thus in male and female simulations we started with an active UGR node as the initial condition, followed by activation of the nodes representing the BPG (CBX2, GATA4, WT1mKTS, WT1pKTS, and NR5A1). The NR0B1, WNT4 and RSPO1 nodes were subsequently activated, in agreement with the reported gene expression patterns, showing that these genes are co-expressed in both male and female embryos during the stage of BPG and previous to GSD (Fig. 3a and b) [7, 34].

If the simulation transited toward the Sertoli attractor, then the NR0B1, WNT4 and RSPO1 nodes were inactivated by NR5A1, SRY, SOX9 and DKK1 nodes. In male the expression of *NROB1* is dosage sensitive, since duplication of this gene in 46,XY patients produces a male-to-female sex reversal with streak gonads [35]. It is important to notice that *NR0B1* might play an important role in male after the time of GSD because *NR0B1* knockout mice showed disorganized Sertoli, Leydig and germ cells due to defects in testis cord formation [36]. Thus, it has been suggested that *NR0B1* has a time frame of expression [37] with reduced levels of the DAX1 protein during GSD. Since the BNM considers active or inactive states, the NR0B1 node was inactive at the sixth time step, which corresponds to the Sertoli attractor (Fig. 3a)

When the UGR + WNT4 nodes were set to ON, two fixed point attractors were obtained: (1) the granulosa attractor and the (2) dysgenetic gonad attractor. Thus the transition towards the granulosa attractor is characterized by the initial activation of the UGR + WNT4 and BPG nodes, followed by activation of the NR0B1, RSPO1, FOXL2 and CTNNB1. As we previously stated, *β*-catenin, plays a key role in up-regulation of pre-granulosa genes in female mouse [32], this factor actively antagonizes *SOX9* and *AMH* expression, inactivating the pathway toward Sertoli cells (Fig. 3b) [38, 39]. The attractor with no activity reflects the importance of the UGR node within the network model given that loss of function mutants of UGR components have an impaired subsequent gonadal development, as observed in mouse. Therefore, the dysgenetic gonad attractor (i.e., streaks of fibrous tissue instead of a gonad) might be interpreted as a condition expected in some individuals when gonadal development fails, especially in the case of *LHX1*, *LHX9*, *EMX2*, *PAX2* and *PAX8* mutants.

In summary, state transitions in Fig. 3a and b qualitatively coincide with gene expression patterns observed during GSD [7, 34]. However, notice that the state transitions and steady state attractors must be considered as snapshots of the gene expression pattern between 41–52 days of development and do not represent the complete process of gonadal development.

### Modeling disorders of sex development

#### 46,XX sex reversal

**Fig. 4:**
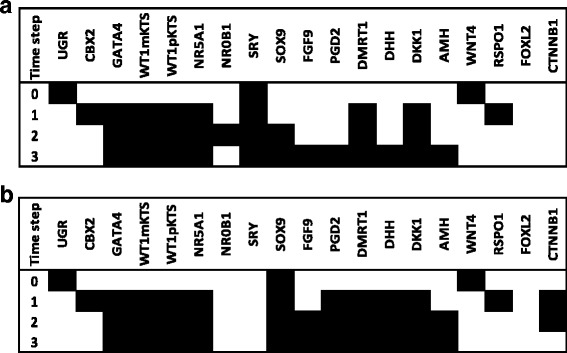
Modeling 46,XX sex reversal. The SRY node was kept as active (1) so as to simulate traslocation in a 46,XX background. SRY activates SOX9 in combination with CBX2, GATA4, WT1 and NR5A1. Then, SOX9 activates nodes associated with the male pathway (i.e., FGF9, PGD2, DHH, AMH). The fixed point attractor can be interpreted as a 46,XX sex reversal after three time steps (**a**). The SOX9 node was set as active in order to simulate a duplication in a 46,XX background (**b**). The resulted activation states were similar to the observed in the male pathway. Thus, the fixed point attractor can be interpreted as a 46,XX sex reversal in absence of SRY, as reported in clinical cases

We simulated the DSD known as 46,XX sex reversal or testicular DSD, characterized by an apparently normal development of male structures, including testes and male internal/external genitalia [20, 40]. To simulate such a condition either the SRY node or the SOX9 node were left permanently active (ON = 1) during the entire simulation (Fig. 4a, b). The SRY node activates SOX9 in coordination with CBX2, GATA4, WT1 and NR5A1, SOX9 in turn inhibits the female pathway through CTNNB1 inactivation. CTNNB1 is the node of the transcription factor *β*-catenin, a key regulatory element of the female pathway. Our BNM generates in both simulations a *Sertoli-like* attractor that presents activation of the FGF9, PGD2, DMRT1, DHH, DKK1 and AMH nodes. The simulation observed in Fig. 4a might be interpreted as the process underlying a 46,XX sex reversal when the *SRY* gene is translocated to one autosomic chromosome or to the X chromosome, whereas the simulation in Fig. 4b might represent a 46,XX sex reversal due SOX9 gene duplication.

#### 46,XY (SRY-) sex reversal

SRY is considered the trigger of the male pathway and testis development [9]. This transcription factor possesses a highly conserved domain called HMG box that binds to the GAACAAAG DNA motif and bends the DNA molecule about 80 degrees. The loss of its chromatin-remodeling activity [41] is considered to impair the three dimensional architecture of chromatin and compromises the proper interaction of SRY with its target genes. Mutation of the DNA binding region of SRY in 46,XY subjects has been associated with female external genitalia, normal Mullerian ducts and streak gonads [42]. When we simulated the SRY loss of function, the female pathway was activated by the CTNNB1 node and the male pathway blocked through a CTNNB1 and FOXL2- mediated *SOX9* inhibition. SRY is considered the trigger of testis development by expressing in the somatic pre-Sertoli cells [9]. The mechanism suggested for normal function of this transcription factor is a highly conserved domain within the protein, called High Mobility Group (HMG box).

**Fig. 5:**
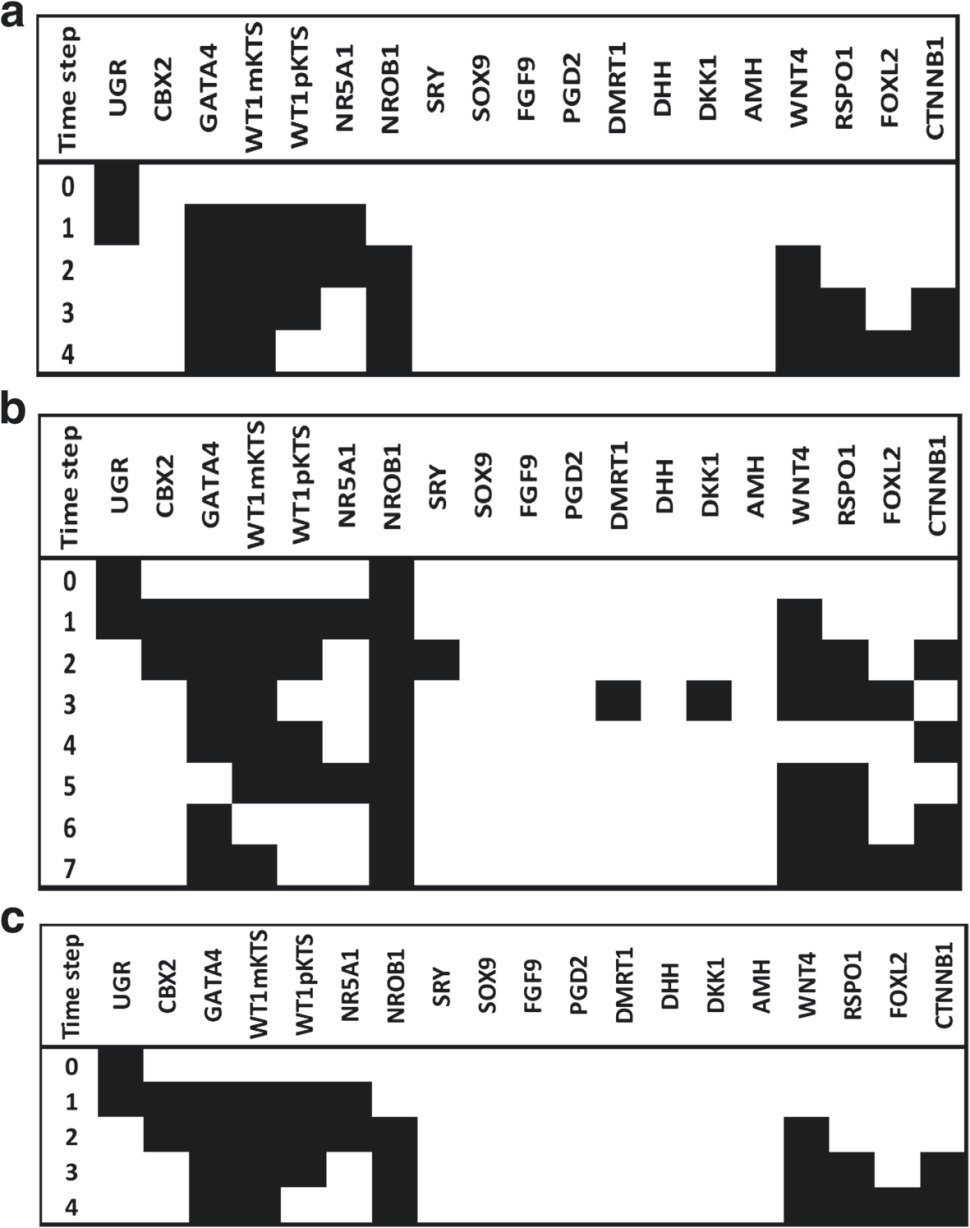
Modeling 46,XY sex reversal. The CBX2 node was inactivated in order to simulate a loss of function mutation in a 46,XY background which resulted in a steady state attractor that showed activation of female nodes (**a**). The NR0B1 node was set as active in order to simulate a duplication in a 46,XY background. The activation states were altered and resulted in an steady state attractor with activated female nodes (**b**). The SRY node was inactivated in order to simulate a loss of function mutation which resulted in a steady state attractor that showed activation of female nodes (**c**)

Concerning the model simulation in Fig. 5c, loss-of-function of SRY leads to inactivation of the male and activation of the female pathway. To this respect, Hawkings and colleagues [42] described five subjects with 46,XY karyotype associated with completely female external genitalia, normal Mullerian ducts, and streak gonads. All the patients showed mutations in the DNA binding region of the SRY protein [42]. Since model simulations agree with clinical observations of loss-of-function mutations in the HMG box of SRY, we interpret this simulation as the possible gene expression dynamics in a 46,XY (SRY-) individual during the time of GSD. In these subjects the female pathway would become active by increasing amounts of *β*-catenin within the cell nucleus and active repression of the male pathway by a B-catenin and FOXL2-mediated inhibition of *SOX9*. Given this results we interpret that our simulations (Fig. 5c) resemble the early gene expression dynamics in a 46,XY (SRY-) individuals.

#### Modeling other DSDs

**Fig. 6:**
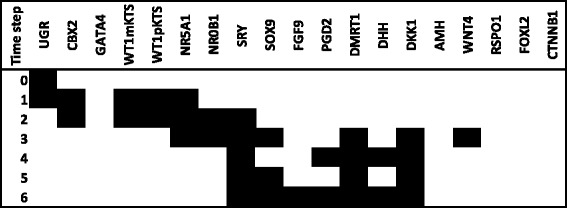
State transitions when the GATA4 node was inactive (0) in a 46,XY context. The GATA4 node was inactivated in order to simulate a loss of function mutation in a 46,XY background which resulted in inactivated AMH node although an attractor with activated male nodes was recovered

Other relevant elements that have a common function in both male and female pathways at the BPG and thorough the differentiation of testis and ovaries are the transcription factors GATA4 and WT1. In the case of GATA4, it has been observed in mice that GATA4 is expressed at E10.5 during formation of the UGR and its deficiency impairs subsequent gonadal differentiation [43]. In the male pathway, GATA4 associates with the -KTS isoform of WT1 protein for an optimal activation of the *SRY* gene [44]. Other example of the GATA4 protein activity in the male pathway is its role in the activation of the *AMH* gene in association with SF1 and WT1-KTS transcription factors [45, 46]. Simulation of GATA4 loss of function in the male pathway is given in Fig. 6 (notice the altered dynamics of activation patterns compared with the *wild-type* simulation shown in Fig. 3a). WT1mKTS, WT1pKTS, NR5A1 and AMH nodes were inactive in the attractor because the GATA4 node is their positive regulator. The SRY node remained active due to an activation self-loop and additional interactions with NR5A1, WT1mKTS, CBX2 and a possible feedback loop with SOX9, thus the altered activation state shown in Fig. 6 might be interpreted as the source of a DSD. To this respect, the clinical spectrum of developmental anomalies due to *GATA4* mutations in male patients is variable. Patients might show bilateral dysgenetic testes containing Sertoli cells and no visible Leydig cells and show male internal genitalia to normal-ambiguous external genitalia [46].

**Fig. 7:**
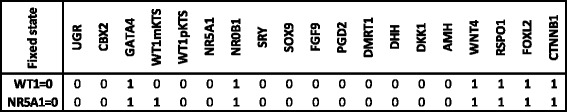
Fixed point attractors when setting NR5A1 or WT1 inactive (0) in a 46,XY context

On the other hand, WT1 has a key role in the development of kidney and gonads, its expression is observed at the UGR and continues through the differentiation of testis and ovaries interacting in both pathways of cell differentiation. Homozygous mutations in mice are embryonic lethal and result in renal agenesis, as well as cardiac and genital tract abnormalities [29]. Since WT1 is an important element in early stages of gonadal development we simulated the loss of function of WT1 in a 46,XY context (Fig. 7), notice that the attractor has an activation pattern similar to the female pathway. Importantly, mutations in this gene impair the development of male in a certain degree; mutations in the intron 9 splice site of the *WT1* gene affects the balance of the +KTS/-KTS isoforms impairing the development of testis resulting in streak gonads and ambiguous external genitalia [47]. In females the role of WT1 is less defined, however there is experimental evidence about its role in the positive regulation of the *NR0B1* gene by binding to two potential sites located at the 5 flanking region of the gene [48].

We analyzed in addition the effect of loss-of-function mutations in the NR5A1 node. Since the dynamics of the simulations of WT1 and NR5A1 were identical we show only the attractor in Fig. 7.

### Testing properties of the Boolean model: random networks and robustness of the attractors

On average, the three independent tests of 1000 random networks generated 103,000 attractors each one. We found that none of the random networks recovered the set of three point attractors of the *wild type* model shown in Fig. 2. This results indicate that the attractors of our BNM could not be expected in a network made with random interactions. Thus, the attractors in Fig. 2 can be considered as biologically meaningful and not a statistical artifact.

Concerning the test of attractor robustness we observed that 95 % of the perturbed networks recovered less than 30 % of the original attractors of our model. This means that the model is relatively sensitive to perturbations in the functions of the model. The reduced robustness can be due to the scarce redundancy in the model, given that we opted by including a small number of regulatory molecules, so as to be close to a minimal model. We expect that the introduction of more nodes and regulatory feedback circuits would result in an increased robustness.

## Discussion

The classical observations of Alfred Jost (1947) that early castration *in utero* of rabbit fetuses resulted in female internal and external genitalia (independent of their chromosomal sex complement) lead to the hypothesis of a testis-determining factor (TDF). According to this hypothesis the ovary was considered the default developmental state, while testis represented an induced and active state that repressed female development. Experimental evidence accumulated in the last 20 years have enriched our view of sex determination where developmental programs toward testis or ovaries represent two independent antagonistic regulatory pathways of high complexity intertwined in a regulatory network: the GSD network.

The BNM used in this study, although discrete in its approach, can be considered as a simplified version of a very dynamic and complex biological network that incorporates the major regulatory elements of the GSD network. The GSD BNM summarizes in a formal language the set of experimentally-confirmed interactions associated with the process of GSD. Although the attractors obtained in our model cannot be interpreted as as anatomical structures of high developmental complexity, they can be reliably seen as the gene expression profiles expected during the process of determination and differentiation of SPC and GPC towards Sertoli and granulosa cells respectively.

### The network of gonadal sex determination

We inferred the regulatory network of human GSD and modeled it as a BNM. With such a model we were able to describe the molecular dynamics of the first stage in gonadal morphogenesis, which is the cell fate determination and further differentiation of Sertoli and granulosa cells. The network contains 19 nodes as well as their regulatory interactions, as evidenced by published experimental and clinical data.

Recent studies regarding early gonadal differentiation suggest a highly complex biological process regulated by many, probably hundreds, of genes. However, our BNM shows that only a handful of them are sufficient to activate the male or female pathway, allowing us to propose that the gonadal fate commitment and differentiation is a direct consequence of activation and repression of a transcriptional program encoded as a regulatory network. Although part of the information used to infer this regulatory network was taken from experiments in mice, we consider that the model might be considered as a good approximation to the corresponding regulatory network in humans.

### The Boolean model of gonadal sex determination

The BNM describes the dynamics of 19 nodes associated with GSD between 41–52 days of embryonic development. The results cannot be considered as final activation states of the biological process, instead they should be considered as a snapshot in the process of Sertoli and granulosa cell differentiation. At the quantitative level, GSD is poorly understood given the lack of information about kinetic details of each regulatory element, therefore it is difficult to establish a continuous model with differential equations with the current available data. Despite the large amount of gene expression data, little is known about the regulatory mechanisms leading to GSD under normal conditions, as well as their downstream effects under mutant conditions. To shed light about the regulatory mechanisms we used a discrete modeling approach because most of the information relies on qualitative descriptions.

### Model predictions and the role of the UGR and bipotential gonad genes in early gonadal development

The UGR is a very important structure within the developing embryo since it is the common structure that leads to testis and ovaries. Notice that just a few regulatory elements of the UGR have been studied. For example, *Lhx1* expression has been reported in mice and has a key role in the development of kidney, female reproductive tract and anterior head [30]. Conditional knockout mice lack uterus, cervix and upper vagina [49]. *Lhx9* has a role in the activation of *Nr5a1* gene, in synergy with *Wt1* in mice. Other example is given by *PAX2* and *PAX8* genes. In humans, these genes have a role in the activation of the *WT1* gene [29, 50] thus, we underline the need for additional studies regarding the regulatory interactions that led to the establishment of the UGR and BPG primordium.

As we mentioned previously, the human female pathway is less characterized, and its current cumulative knowledge is mainly based on mice findings. In this case, the GATA4-FOG2 complex has an important function by activating the *Fst*, *Wnt4*, *Sprr2d*, *Foxl2*, *Gng13* genes [32]. It has been observed in female mice that loss of function of *Gata4* impairs the expression of these genes and leads to the development of a male-specific coelomic vessel [32]; therefore GATA4 can be considered as a key regulatory element in the early stages of gonadal development toward ovaries.

### The role of WNT4/ *β*-catenin in the female pathway

Concerning the female pathway most of the inferred interactions derive from mice. To this respect we notice the role of the WNT4 and *β*catenin nodes in the regulation of WNT4, RSPO1, and FOXL2. In the biological process we predict a key role of *β*-catenin regulating the female pathway. The general mechanism of the canonical Wnt4/ *β*-catenin signaling pathway can be explained as follows: the pathway is initiated by expression of the wingless-type MMTV integration site family, member 4 (*WNT4*). The product of this gene is a ligand that binds to Frizzled (Fz) and LRP5/6 co-receptors at the plasma membrane, disengaging *β*-catenin from the proteins of the “destruction complex”? (Axin and APC). Then *β*-catenin translocate into the nucleus where associates with TCF7/LEF, this protein contains an HMG box with capacity to recognize specific DNA sequences. The *β*-catenin-TCF7/LEF complex activates target genes, whereas in absence of *β*-catenin TCF7/LEF alone represses gene transcription [51, 52]. From the set of interactions inferred from mice, that fitted perfectly in our model, we would expect that *β*-catenin might have an important role regulating the expression of the *FST*, *FOXL2* and *IRX3* genes in humans.

The BNM of GSD summarizes in a formal language the set of experimentally-confirmed interactions associated with the process of GSD. The attractors of our model can be interpreted as the gene expression profiles expected during the process of GSD and differentiation of Sertoli or granulosa cell lineages. According to our simulations, the loss of function of GATA4 results in inactivation of the AMH node in the attractor. This result is particularly interesting given the existence of the persistent Müllerian duct syndrome (PMDS), a relatively rare inherited defect in the sexual differentiation, characterized by failure in the regression of the Müllerian ducts in males. Affected individuals present persistent uterus and tubes due to a defect in the synthesis of the AMH hormone, which is normally produced by the Sertoli cells. Mutations in the AMH gene have been reported in these patients [20, 53], however the majority of PMDS remain without molecular diagnosis, therefore GATA4 mutations emerge, according to our model predictions, as a potential PMSD causing gene.

## Conclusions

We propose the present model as a starting point for future mathematical modeling and integration of experimental research regarding sex development. The model can be upgraded in several aspects for example, incorporating additional nodes and interactions, as well as modeling more cell lineages of the gonad such as the Leydig or theca cells. Finally the current BNM describes the dynamics of the GSD network under perturbations. Importantly the analysis of these states can have potential implications in the study of DSDs.

## Additional file

Additional file 1
**Supplementary information.** (PDF 47 kb)
