# Supplementary material for: A Boolean network model of human gonadal sex determination
Source: Theor Biol Med Model. 2015 Nov 16;12:26. doi: 10.1186/s12976-015-0023-0 (PMC4647291; doi:10.1186/s12976-015-0023-0)
Supplement: Additional file 1 — Supplementary information. (PDF 47 kb) [file 12976_2015_23_MOESM1_ESM.pdf]

Supplementary information

November 4, 2015

Table 1: Regulatory interactions used to construct the Boolean model of gonadal sex determination.

Interaction	Species	References
Emx2→ Urogenital ridge	*Mus musculus*	[1, 2]
LHX1→ Urogenital ridge	*Homo sapiens*	[3]
LHX9 + WT1mKTS→ *NR5A1*	*Homo sapiens*	[4, 2]
PAX2→ *WT1*WT1	*Homo sapiens*	[5, 6]
PAX8→ *WT1*	*Homo sapiens*	[7]
CBX2→ *NR5A1*	*Homo sapiens*	[8]
CBX2→ *SRY*	*Homo sapiens*	[8]
CBX2→ *SOX9*	*Homo sapiens*	[8]
GATA4/FOG2 + SF1→ *AMH*	*Homo sapiens*	[9, 10, 11, 12]
GATA4 + FOG2→ *SRY*	*Homo sapiens*	[13, 14]
GATA4 + WT1pKTS→ *SRY*	*Homo sapiens*	[15]
Gata4→ *Wnt4*	*Mus musculus*	[16]
WT1mKTS→ *NR5A1*	*Homo sapiens*	[17, 4]
WT1mKTS→ *NR0B1*	*Homo sapiens*	[18]
WT1mKTS→ *SRY*	*Homo sapiens*	[19, 20]
WT1mKTS + SF1→ *AMH*	*Homo sapiens*	[19, 17, 15]
WT1mKTS + SF1→ *SRY*	*Homo sapiens*	[21, 22, 23]
SF1→ *NR0B1*	*Homo sapiens*	[24, 25, 26, 27]
DAX1⊣ *SF1*	*Homo sapiens*	[24, 28, 29]
DAX1⊣ SF1 + GATA4 + WT1 → *AMH*	*Homo sapiens*	[30, 17]
SRY+SF1→ *SOX9*	*Homo sapiens*	[31, 32]
SRY→ *SOX9*	*Homo sapiens*	[31, 32]
SRY⊣ β-catenin	*Homo sapiens*	[33]
Sox9→ *Sry*	*Mus musculus*	[34, 35]
Sox9→ *Fgf9*	*Mus musculus*	[36]
SOX9→ *PGD2*	*Homo sapiens*	[37, 38]
SOX9 + SF1→ *AMH*	*Homo sapiens*	[39, 40]
SOX9⊣ β-catenin	*Homo sapiens*	[33]
SOX9⊣ FOXL2	*Homo sapiens*	[41]
Fgf9→ *Sox9*	*Mus musculus*	[36, 42, 43]
Fgf9⊣ Wnt4	*Mus musculus*	[43]
PGD2→ *SOX9*	*Homo sapiens*	[37, 38]
Dkk1⊣ Wnt4-β-catenin	*Mus musculus*	[16]
Continues ...		

Continuation		
Interaction	Species	References
Dkk1$\dashv$ Rspo1	*Mus musculus*	[16]
WNT4$\rightarrow$ NR0B1	*Homo sapiens*	[44]
WNT4$\dashv$ SF1	*Homo sapiens*	[45]
Wnt4$\dashv$ Fgf9	*Mus musculus*	[43]
Wnt4$\dashv$ Sox9	*Mus musculus*	[43]
RSPO1$\rightarrow$ Wnt4-β-catenin	*Homo sapiens*	[46, 33]
FOXL2$\dashv$ SF1 $\rightarrow$ CYP17	*Homo sapiens*	[47]
FOXL2$\dashv$ SOX9	*Homo sapiens*	[41]
FOXL2$\dashv$ DMRT1	*Homo sapiens*	[48]
β-catenin$\rightarrow$ Foxl2	*Mus musculus*	[16]
β-catenin$\dashv$ Sry, Sox9, Amh	*Mus musculus*	[49]

The genes and protein names were written in human an mouse according to international standard nomenclature

http://www.genenames.org/about/faq

http://www.informatics.jax.org/mgihome/nomen/gene.shtml

References

[1] Pellegrini M, Pantano S, Lucchini F, Fumi M, Forabosco A. *Emx2* developmental expression in the primordia of the reproductive and excretory systems. Anat Embryol. 1997;196(6):427-433.

[2] Ostrer H, Huang HY, Masch RJ, Shapiro E. A cellular study of human testis development. Sex Dev. 2007;1(5):286-292.

[3] Kobayashi A, Shawlot W, Kania A, Behringer, RR. Requirement of Lim1 for female reproductive tract development. Development. 2004;131(3): 539-549.

[4] Wilhelm D, Englert C. The Wilms tumor suppressor WT1 regulates early gonad development by activation of Sf1. Genes & Development. 2002;16(14):1839-1851.

[5] McConnell MJ, Cunliffe HE, Chua LJ, Ward, TA, Eccles MR. Differential regulation of the human Wilms tumour suppressor gene (WT1) promoter by two isoforms of PAX2. Oncogene. 1997;14(22):2689-2700.

[6] Torres M, Gómez-Pardo E, Dressler GR, Gruss P. Pax-2 controls multiple steps of urogenital development. Development. 1995;121(12):4057-4065.

[7] Fraizer GC, Shimamura R, Zhang X, Saunders GF. PAX 8 regulates human WT1 transcription through a novel DNA binding site. J Biol Chem. 1997;272(49):30678-30687.

[8] Biason-Lauber A, Konrad D, Meyer M, Schoenle EJ. Ovaries and female phenotype in a girl with 46,XY karyotype and mutations in the CBX2 gene. Am J Hum Genet. 2009;84(5):658-663.

[9] Tremblay JJ, Viger RS. Transcription factor GATA-4 enhances Müllerian inhibiting substance gene transcription through a direct interaction with the nuclear receptor SF-1. Mol Endocrinol.1999;13(8):1388-1401.

[10] Tremblay JJ, Robert NM, Viger RS. Modulation of endogenous GATA-4 activity reveals its dual contribution to Müllerian inhibiting substance gene transcription in Sertoli cells. Mol Endocrinol. 2001;15(9):1636-1650.

[11] Tremblay JJ, Viger RS. GATA Factors Differentially Activate Multiple Gonadal Promoters through Conserved GATA Regulatory Elements 1. Endocrinology. 2001;142(3):977-986.

[12] White S, Ohnesorg T, Notini A, Roeszler K, Hewitt J, Daggag H. Copy number variation in patients with disorders of sex development due to 46,XY gonadal dysgenesis. PLoS ONE. 2011;6(3):e17793.

[13] Lourenço D, Brauner R, Rybczyńska M, Nihoul-Fékété, C, McElreavey K, Bashamboo A. Loss-of-function mutation in GATA4 causes anomalies of human testicular development. P. Natl. Acad. Sci. 2011;108(4):1597-1602.

[14] Su H, Lau YF. Identification of the transcriptional unit, structural organization, and promoter sequence of the human sex-determining region Y (SRY) gene, using a reverse genetic approach. Am J Hum Genet. 1993;52(1):24

[15] Miyamoto Y, Taniguchi H, Hamel F, Silversides DW, Viger RS. A GATA4/WT1 cooperation regulates transcription of genes required for mammalian sex determination and differentiation. BMC Mol Biol. 2008;9(1):44.

[16] Manuylov NL, Smagulova FO, Leach L, Tevosian SG. Ovarian development in mice requires the GATA4-FOG2 transcription complex. Development. 2008;135(22):3731-3743.

[17] Nachtigal MW, Hirokawa Y, Enyeart-VanHouten DL, Flanagan JN, Hammer GD, Ingraham HA. Wilms' tumor 1 and Dax-1 modulate the orphan nuclear receptor SF-1 in sex-specific gene expression. Cell. 1998;93(3):445-454.

[18] Kim J, Prawitt D, Bardeesy N, Torban E, Vicaner, C, Goodyer P. The Wilms tumor suppressor gene (wt1) product regulates Dax-1 gene expression during gonadal differentiation. Mol Cell Biol. 1999;19(3):2289-2299.

[19] Shimamura R, Fraizer GC, Trapman J, Lau YFC, Saunders GF. The Wilms'tumor gene WT1 can regulate genes involved in sex determination and differentiation: SRY, Müllerian-inhibiting substance, and the androgen receptor. Clin Cancer Res. 1997;3(12):2571-2580.

[20] Hersmus R, Van der Zwan YG, Stoop H, Bernard P, Sreeniwasan R, Oosteerhuis JW. A 46, XY female DSD patient with bilateral gonadoblastoma, a novel SRY missense mutation combined with a WT1 KTS splice-site mutation. PloS ONE. 2012;7(7): e40858-e40858.

[21] Achermann, JC, Ito M, Ito M, Hindmarsh PC, Jameson JL. A mutation in the gene encoding steroidogenic factor-1 causes XY sex reversal and adrenal failure in humans. Nat Genet. 1999;22(2):125-126.

[22] de Santa Barbara P, Mejean C, Moniot B, Malcls MH, Berta P, Boizet-Bonhoure B. Steroidogenic factor-1 contributes to the cyclic-adenosine monophosphate down-regulation of human SRY gene expression. Biol Reprod. 2001;64(3):775-783.

[23] Tantawy S, Mazen I, Soliman H, Anwar G, Atef A, El-Gammal M. Analysis of the gene coding for steroidogenic factor 1 (SF1, NR5A1) in a cohort of 50 Egyptian patients with 46, XY disorders of sex devellopment. Eur J Endocrinol. 2014;170(5):759-767.

[24] Achermann JC, Meeks JJ, Jameson JL. Phenotypic spectrum of mutations in DAX-1 and SF-1. Mol Cell Endocrinol. 2001;185(1):17-25.

[25] Burris TP, Guo WW, Le T, McCabe ER. Identification of a putative steroidogenic factor-1 response element in the DAX-1 promoter. Biochem Bioph Res Co. 1995;214(2):576-581.

[26] Mizusaki H, Kawabe K, Mukai T, Ariyoshi E, Kasahara M, Yoshioka H. Dax-1 (dosage-sensitive sex reversal-adrenal hypoplasia congenita critical region on the X chromosome, gene 1) gene transcription is regulated by wnt4 in the female developing gonad. Mol Endocrinol. 2003;17(4): 507-519.

[27] Vilain E, Guo W, Zhang YH, McCabe ER. DAX1 gene expression upregulated by steroidogenic factor 1 in an adrenocortical carcinoma cell line. Biochem Mol Med. 1997;61(1):1-8.

[28] Crawford PA, Dorn C, Sadovsky Y, Milbrandt J. Nuclear receptor DAX-1 recruits nuclear receptor corepressor N-CoR to steroidogenic factor 1. Mol Cell Bio. 1998;18(5):2949-2956.

[29] Ito M, Yu R, Jameson, JL. DAX-1 inhibits SF-1-mediated transactivation via a carboxy-terminal domain that is deleted in adrenal hypoplasia congenita. Mol Cell Bio.1997;17(3):1476-1483.

[30] Tremblay JJ, Viger RS. Nuclear receptor Dax-1 represses the transcriptional cooperation between GATA-4 and SF-1 in Sertoli cells. Biol Reprod. 2001;64(4):1191-1199.

[31] Sekido R, Lovell-Badge R. Sex determination involves synergistic action of SRY and SF1 on a specific Sox9 enhancer. Nature. 2008;453(7197):930-934.

[32] Knower KC, Kelly S, Ludbrook LM, Bagheri-Fam S, Sim, H. Bernard P et al. Failure of SOX9 regulation in 46XY disorders of sex development with SRY, SOX9 and SF1 mutations. PLoS ONE. 2011;6(3):e17751.

[33] Bernard P, Sim H, Knower K, Vilain E, Harley V. Human SRY inhibits β-catenin-mediated transcription. Int J Biochem Cell B. 2008;40(12):2889-2900.

[34] Daneau I, Pilon N, Boyer A, Behdjani R, Overbeek PA., Viger R, et al. The porcine SRY promoter is transactivated within a male genital ridge environment. Genesis. 2002;33(4):170-180.

[35] Ross DG, Bowles J, Koopman P, Lehnert S. New insights into SRY regulation through identification of 5'conserved sequences. BMC Mol Biol. 2008;9(1):85

[36] Colvin, JS, Green RP, Schmahl J, Capel B, Ornitz DM. Male-to-female sex reversal in mice lacking fibroblast growth factor 9. Cell. 2001;104(6): 875-889.

[37] Wilhelm D, Hiramatsu R, Mizusaki H, Widjaja L. Combes AN, Kanai Y. SOX9 regulates prostaglandin D synthase gene transcription in vivo to ensure testis development. J Biol Chem. 2007;282(14):10553-10560.

[38] Moniot B, Declosmenil F, Barrionuevo F, Scherer G, Aritake K, Malki S. The PGD2 pathway, independently of FGF9, amplifies SOX9 activity in Sertori cells during male sexual differentiation. Development. 2009; 136(11):1813-1821.

[39] De Santa Barbara P, Bonneaud N, Boizet B, Desclozeaux M, Moniot B, et al. Direct interaction of SRY-related protein SOX9 and steroidogenic factor 1 regulates transcription of the human anti-Müllerian hormone gene. Mol Cell Biol. 1998;18(11):6653-6665.

[40] De Santa Barbara P, Moniot B, Poulat F, Berta P. Expression and subcellular localization of SF-1,SOX9, WT1, and AMH proteins during early human testicular development. Dev Dynam. 2000;217(3):293-298.

[41] Hersmus R, Kalfa N, de Leeuw B, Stoop H, Oosterhuis JW, de Krijeer R. FOXL2 and SOX9 as parameters of female and male gonadal differentiation in patients with various forms of disorders of sex development (DSD). The Journal of pathology. 2008;215(1):31-38.

[42] Schmahl J, Kim Y, Colvin JS, Ornitz DM, Capel B. *Fgf9* induces proliferation and nuclear localization of FGFR2 in Sertoli precursors during male sex determination. Development. 131;(15):3627-3636.

[43] Kim Y, Kobayashi A, Sekido R, DiNapoli L, Brennan J, Chaboissier, MC, et al. Fgf9 and Wnt4 act as antagonistic signals to regulate mammalian sex determination. PLoS Biol. 2006;4(6):e187.

[44] Jordan BK, Mohammed M, Ching ST, Déllot E, Chen XN, Dewing P. Up-regulation of WNT-4 signaling and dosage-sensitive sex reversal in humans. Am J Hum Genet. 2001;68(5):1102-1109.

[45] Jordan BK, Shen JHC, Olaso R, Ingraham HA, Vilain E. Wnt4 overexpression disrupts normal testicular vasculature and inhibits testosterone synthesis by repressing steroidogenic factor 1/β-catenin synergy. P. Natl. Acad. of Sci. 2003;100(19):10866-10871.

[46] Tomaselli S, Megiorni F, Lin L, Mazzilli MC, Gerrelli D, Majore S, Grammatico P, Achermann JC. Human RSPO1/R-spondin1 is expressed during early ovary development and augments β-catenin signaling. PLoS ONE. 2011;6(1):e16366.

[47] Park M, Shin E, Won M, Kim JH, Go H, Kim HL. FOXL2 interacts with steroidogenic factor-1 (SF-1) and represses SF-1-induced CYP17 transcription in granulosa cells. Mol Endocrinol. 2010; 24(5):1024-1036.

[48] Matson CK, Murphy MW, Sarver AL, Griswold MD, Bardwell WJ, Zarkower D. DMRT1 prevents female reprogramming in the postnatal mammalian testis. Nature. 2011;476(7358):101-104.

[49] Maatouk DM, DiNapoli L, Alvers A, Parker KL, Taketo MM, Capel B. Stabilization of β-catenin in XY gonads causes male-to-female sex-reversal. Hum Mol Genet. 2008;17(19): 2949-2955.
